# Dicobalt(II) lead(II) hydrogenphos­phate(V) phos­phate(V) hydroxide monohydrate

**DOI:** 10.1107/S1600536812014870

**Published:** 2012-04-18

**Authors:** Abderrazzak Assani, Mohamed Saadi, Mohammed Zriouil, Lahcen El Ammari

**Affiliations:** a Laboratoire de Chimie du Solide Appliquée, Faculté des Sciences, Université Mohammed V-Agdal, Avenue Ibn Battouta, BP 1014, Rabat, Morocco

## Abstract

The title compound, Co_2_Pb(HPO_4_)(PO_4_)OH·H_2_O, which was synthesized under hydro­thermal conditions, crystallizes in a new structure type. Except for two O atoms in general positions and two Co atoms on centres of symmetry, all other atoms in the asymmetric unit (1 Pb, 2 Co, 2 P, 8 O and 4 H) are located on mirror planes. The structure is built up from two infinite linear chains, *viz.* ^1^_∞_[CoO_2/1_(H_2_O)_2/2_O_2/2_] and ^1^_∞_[CoO_2/1_(OH)_2/2_O_2/2_], of edge-sharing CoO_6_ octa­hedra running along [010]. Adjacent chains are linked to each other through PO_4_ and PO_3_(OH) tetra­hedra, leading to the formation of layers parallel to (100). The three-dimensional framework is formed by stacking along [100] of adjacent layers that are held together by distorted PbO_8_ polyhedra. Hydrogen bonds of the type O—H⋯O involving the water mol­ecule are very strong, while those O atoms involving the OH groups form weak bifurcated and trifurcated hydrogen bonds.

## Related literature

For catalytic properties of phosphates, see: Cheetham *et al.* (1999 ▶); Clearfield (1988 ▶); Trad *et al.* (2010 ▶). For compounds with related structures, see: Yakubovich *et al.* (2001 ▶); Lee *et al.* (2008 ▶); Effenberger (1999 ▶); Britvin *et al.* (2002 ▶); Assani *et al.* (2010 ▶). For bond-valence analysis, see: Brown & Altermatt (1985 ▶). For background to the Inorganic Crystal Structure Database (ICSD), see: Belsky *et al.* (2002 ▶).

## Experimental

### 

#### Crystal data

Co_2_Pb(HPO_4_)(PO_4_)OH·H_2_O
*M* *_r_* = 551.02
Monoclinic, 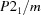

*a* = 7.4299 (1) Å
*b* = 6.2949 (1) Å
*c* = 8.9057 (1) Å
β = 113.936 (1)°
*V* = 380.70 (1) Å^3^
*Z* = 2
Mo *K*α radiation
μ = 26.83 mm^−1^
*T* = 296 K
0.18 × 0.12 × 0.08 mm

#### Data collection

Bruker X8 APEXII diffractometer
Absorption correction: multi-scan (*SADABS*; Sheldrick, 1999 ▶) *T*_min_ = 0.029, *T*_max_ = 0.117
8321 measured reflections
1601 independent reflections
1558 reflections with *I* > 2σ(*I*)
*R*_int_ = 0.027

#### Refinement

*R*[*F* ^2^ > 2σ(*F* ^2^)] = 0.016
*wR*(*F* ^2^) = 0.039
*S* = 1.11
1601 reflections
86 parameters
H-atom parameters constrained
Δρ_max_ = 1.76 e Å^−3^
Δρ_min_ = −1.49 e Å^−3^

### 

Data collection: *APEX2* (Bruker, 2005 ▶); cell refinement: *SAINT* (Bruker, 2005 ▶); data reduction: *SAINT*; program(s) used to solve structure: *SHELXS97* (Sheldrick, 2008 ▶); program(s) used to refine structure: *SHELXL97* (Sheldrick, 2008 ▶); molecular graphics: *ORTEP-3 for Windows* (Farrugia, 1997 ▶) and *DIAMOND* (Brandenburg, 2006 ▶); software used to prepare material for publication: *PLATON* (Spek, 2009 ▶) and *publCIF* (Westrip, 2010 ▶).

## Supplementary Material

Crystal structure: contains datablock(s) global. DOI: 10.1107/S1600536812014870/wm2609sup1.cif

Additional supplementary materials:  crystallographic information; 3D view; checkCIF report

## Figures and Tables

**Table 1:** Hydrogen-bond geometry (Å, °)

*D*—H⋯*A*	*D*—H	H⋯*A*	*D*⋯*A*	*D*—H⋯*A*
O2—H2⋯O6^i^	0.86	2.28	3.027 (3)	145
O2—H2⋯O6^ii^	0.86	2.28	3.027 (3)	145
O7—H7⋯O2^iii^	0.86	2.20	2.915 (3)	140
O7—H7⋯O3	0.86	2.53	2.986 (2)	114
O7—H7⋯O3^iv^	0.86	2.53	2.986 (2)	114
O8—H8*A*⋯O4^v^	0.86	1.79	2.649 (3)	177
O8—H8*B*⋯O7	0.86	1.65	2.489 (3)	165

Symmetry codes: (i) 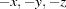; (ii) 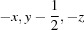; (iii) 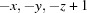; (iv) 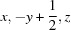; (v) 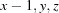.

**Fig. 1.**
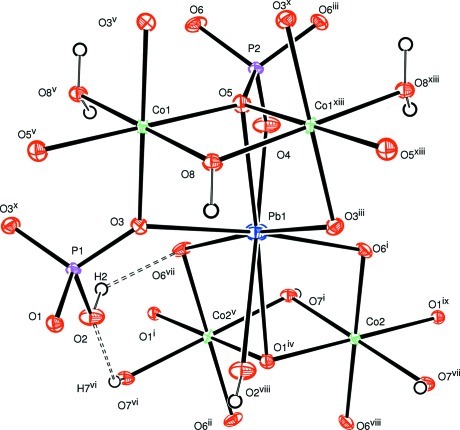
A partial three-dimensional plot of the crystal structure of Co2Pb(HPO4)(PO4)OH.H2O. Displacement ellipsoids are drawn at the 50% probability level. Symmetry codes:(i) -x, y + 1/2, -z; (ii) -x, -y, -z; (iii) x, -y + 1/2, z; (iv) -x, -y, -z + 1; (v) -x - 1, -y, -z; (vi) x + 1, y, z; (vii) -x, -y + 1, -z + 1; (viii) x + 1, -y + 1/2, z + 1; (ix) x + 1, y + 1, z; (x) x, -y - 1/2, z; (xi) x - 1, y - 1, z; (xii) -x, y - 1/2, -z + 1; (xiii) -x - 1, y + 1/2, -z;

**Fig. 2.**
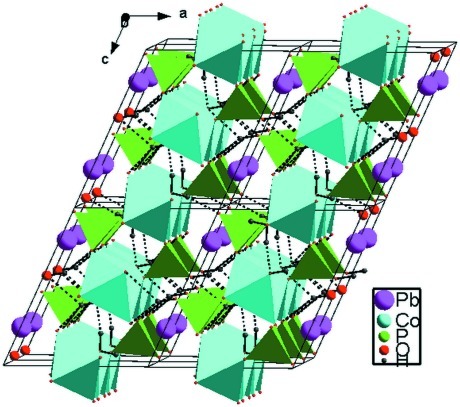
A three-dimensional polyhedral view of the crystal structure of Co2Pb(HPO4)(PO4)OH.H2O, showing the stacking of layers along the [100] axis and the hydrogen bonding scheme (dashed lines).

## supplementary crystallographic information

### Comment

Metal based phosphates are of great interest owing to either their remarkable diversity of structures or their properties and applications in catalysis, as ion-exchangers (Cheetham *et al.*, 1999; Clearfield, 1988) or as positive electrode materials in lithium- and sodium-containing batteries (Trad *et al.*, 2010)). Mainly, our focus of investigation is focused on orthophosphates with (mixed) divalent metals with general formula (*M*,*M*')_3_(PO_4_)_2_^.^*n*H_2_O (Assani *et al.*, 2010). It has been pointed out that the structural diversity of this family of compounds depends on the size difference of the divalent cations (Effenberger, 1999) and on the degree of hydratation (Yakubovich *et al.*, 2001; Lee *et al.*, 2008. The highest water content known up to date is realised for Mg_3_(PO_4_)_2_^.^22H_2_O (Britvin *et al.*, 2002). In this work, a new dicobalt lead phosphate(V)] with formula Co_2_Pb(HPO_4_)(PO_4_)OH^.^H_2_O, was hydrothermally synthesized and structurally characterized.

A search in the ICSD (Belsky *et al.*, 2002) reveals that the crystal structure of this phosphate represents a new structure type. A plot of the crystal structure illustrating the most important coordination polyhedra and their mutual connections is represented in Fig. 1. All atoms are in special positions, except two oxygen atoms (O3,O6) in general position of the *P*2_1_/*m* space group. The crystal structure is built up from three different types of polyhedra more or less distorted, *viz*. two PbO_8_ polyhedra (*m* symmetry), PO_4_ and PO_3_(OH) tetrahedra (both with *m* symmetry) and two CoO_6_ octahedra (both with 1 symmetry). The CoO_6_ octahedra share edges and form ^1^_∞_[Co(1)O_2/1_(H_2_O)_2/2_O_2/2_] and ^1^_∞_[Co(2)O_2/1_(OH)_2/2_O_2/2_] chains running parallel to [010], as shown in Fig. 2. Adjacent chains are connected by PO_4_ and HPO_4_ tetrahedra *via* vertices, leading to the formation of layers parallel to (100). These layers are in turn linked by sheets of distorted PbO_8_ polyhedra as also shown in Fig.2.

Bond valence sum calculations (Brown & Altermatt, 1985) for Pb1^2+^, Co1^2+^, Co2^2+^, P1^5+^ and P2^5+^ ions are as expected, *viz*. 1.92, 2.03, 1.93, 5.05 and 5.05 valence units, respectively. The values of the bond valence sums calculated for the oxygen atoms show low values for O2, O7 and O8 when the contribution of H atoms are not considered (i.e. 1.23, 0.88 and 0.75 valence units, respectively). Hence these O atoms are associated with protons and are involved in O—H···O hydrogen bonding (Table 1). H atoms of the water molecule form very strong hydrogen bonds, especially O8–H8B···O7 with an *D*···*A* distance less than 2.5 Å. The H atom of the OH^-^ group (O7) and the hydrogenphosphate group (O2) form weak bifurcated (O2) and trifurcated (O7) hydrogen bonds (Fig. 2, Table 1).

### Experimental

The title compound, Co_2_Pb(HPO_4_)(PO_4_)OH^.^H_2_O, was obtained from the hydrothermal treatment of a reaction mixture of Pb(NO_3_)_2_, metallic cobalt and 85_wt_% phosphoric acid in the molar ratio Pb:Co:P = 1:3:3. The hydrothermal reaction was conducted in a 23 ml Teflon-lined autoclave under autogeneous pressure at 473 K for three days. The product was filtered off, washed with deionized water and air dried. The resulting product consists of pink crystals besides some pink powder.

### Refinement

The O-bound H atoms were initially located in a difference map and refined with O—H distance restraints of 0.86 (1). In a the last cycle they were refined in the riding model approximation with *U*_iso_(H) set to 1.2*U*_eq_(O). The highest remaining positive and negative electron densities observed in the final Fourier map are 0.76 Å and 0.78 Å, respectively, from Pb1.

### Crystal data

**Table:**

Co_2_Pb(HPO_4_)(PO_4_)OH·H_2_O	*F*(000) = 500
*M**_r_* = 551.02	*D*_x_ = 4.807 Mg m^−^^3^
Monoclinic, *P*2_1_/*m*	Mo *K*α radiation, λ = 0.71073 Å
Hall symbol: -P 2yb	Cell parameters from 1601 reflections
*a* = 7.4299 (1) Å	θ = 2.5–33.5°
*b* = 6.2949 (1) Å	µ = 26.83 mm^−^^1^
*c* = 8.9057 (1) Å	*T* = 296 K
β = 113.936 (1)°	Prism, pink
*V* = 380.70 (1) Å^3^	0.18 × 0.12 × 0.08 mm
*Z* = 2	

### Data collection

**Table:**

Bruker X8 APEXII diffractometer	1601 independent reflections
Radiation source: fine-focus sealed tube	1558 reflections with *I* > 2σ(*I*)
Graphite monochromator	*R*_int_ = 0.027
φ and ω scans	θ_max_ = 33.5°, θ_min_ = 2.5°
Absorption correction: multi-scan (*SADABS*; Sheldrick, 1999)	*h* = −9→11
*T*_min_ = 0.029, *T*_max_ = 0.117	*k* = −9→9
8321 measured reflections	*l* = −13→13

### Refinement

**Table:**

Refinement on *F*^2^	Secondary atom site location: difference Fourier map
Least-squares matrix: full	Hydrogen site location: difference Fourier map
*R*[*F*^2^ > 2σ(*F*^2^)] = 0.016	H-atom parameters constrained
*wR*(*F*^2^) = 0.039	*w* = 1/[σ^2^(*F*_o_^2^) + (0.0153*P*)^2^ + 0.885*P*] where *P* = (*F*_o_^2^ + 2*F*_c_^2^)/3
*S* = 1.11	(Δ/σ)_max_ = 0.001
1601 reflections	Δρ_max_ = 1.76 e Å^−^^3^
86 parameters	Δρ_min_ = −1.49 e Å^−^^3^
0 restraints	Extinction correction: *SHELXL97* (Sheldrick, 2008), Fc^*^=kFc[1+0.001xFc^2^λ^3^/sin(2θ)]^-1/4^
Primary atom site location: structure-invariant direct methods	Extinction coefficient: 0.0061 (3)

### Special details

**Table:**

Geometry. All e.s.d.'s (except the e.s.d. in the dihedral angle between two l.s. planes) are estimated using the full covariance matrix. The cell e.s.d.'s are taken into account individually in the estimation of e.s.d.'s in distances, angles and torsion angles; correlations between e.s.d.'s in cell parameters are only used when they are defined by crystal symmetry. An approximate (isotropic) treatment of cell e.s.d.'s is used for estimating e.s.d.'s involving l.s. planes.
Refinement. Refinement of *F*^2^ against all reflections. The weighted *R*-factor *wR* and goodness of fit *S* are based on *F*^2^, conventional *R*-factors *R* are based on *F*, with *F* set to zero for negative *F*^2^. The threshold expression of *F*^2^ > 2σ(*F*^2^) is used only for calculating *R*-factors(gt) *etc*. and is not relevant to the choice of reflections for refinement. *R*-factors based on *F*^2^ are statistically about twice as large as those based on *F*, and *R*- factors based on all data will be even larger.

### Fractional atomic coordinates and isotropic or equivalent isotropic displacement parameters (Å^2^)

**Table:**

	*x*	*y*	*z*	*U*_iso_*/*U*_eq_	
Pb1	0.006473 (17)	0.2500	0.232477 (15)	0.01414 (5)	
Co1	−0.5000	0.0000	0.0000	0.00717 (8)	
Co2	0.5000	0.5000	0.5000	0.00859 (8)	
P1	−0.22391 (12)	−0.2500	0.32517 (9)	0.00646 (13)	
P2	−0.21025 (11)	0.2500	−0.16270 (9)	0.00642 (13)	
O1	−0.3205 (3)	−0.2500	0.4478 (3)	0.0088 (4)	
O2	0.0058 (4)	−0.2500	0.4403 (3)	0.0145 (5)	
H2	0.0642	−0.2500	0.3744	0.017*	
O3	−0.2656 (2)	−0.0469 (3)	0.2228 (2)	0.0102 (3)	
O4	0.0041 (4)	0.2500	−0.0355 (3)	0.0160 (5)	
O5	−0.3411 (3)	0.2500	−0.0638 (3)	0.0096 (4)	
O6	−0.2489 (3)	0.0525 (3)	−0.2742 (2)	0.0135 (3)	
O7	−0.4341 (4)	0.2500	0.3936 (3)	0.0100 (4)	
H7	−0.3197	0.2500	0.3910	0.012*	
O8	−0.6059 (3)	0.2500	0.0885 (3)	0.0086 (4)	
H8A	−0.7322	0.2500	0.0522	0.010*	
H8B	−0.5660	0.2500	0.1937	0.010*	

### Atomic displacement parameters (Å^2^)

**Table:**

	*U*^11^	*U*^22^	*U*^33^	*U*^12^	*U*^13^	*U*^23^
Pb1	0.01157 (7)	0.02066 (8)	0.01059 (7)	0.000	0.00492 (4)	0.000
Co1	0.00890 (17)	0.00553 (19)	0.00703 (16)	0.00041 (14)	0.00319 (14)	0.00011 (13)
Co2	0.01066 (18)	0.0070 (2)	0.00765 (16)	−0.00114 (14)	0.00323 (14)	−0.00053 (13)
P1	0.0091 (3)	0.0062 (3)	0.0048 (3)	0.000	0.0035 (3)	0.000
P2	0.0068 (3)	0.0075 (3)	0.0052 (3)	0.000	0.0028 (2)	0.000
O1	0.0122 (10)	0.0082 (10)	0.0084 (9)	0.000	0.0067 (8)	0.000
O2	0.0080 (10)	0.0233 (14)	0.0106 (10)	0.000	0.0022 (8)	0.000
O3	0.0119 (7)	0.0090 (7)	0.0083 (6)	−0.0011 (6)	0.0027 (5)	0.0010 (6)
O4	0.0073 (10)	0.0287 (15)	0.0101 (10)	0.000	0.0016 (8)	0.000
O5	0.0110 (10)	0.0101 (10)	0.0105 (9)	0.000	0.0071 (8)	0.000
O6	0.0156 (8)	0.0124 (8)	0.0088 (6)	0.0056 (7)	0.0010 (6)	−0.0041 (6)
O7	0.0113 (10)	0.0130 (11)	0.0074 (9)	0.000	0.0055 (8)	0.000
O8	0.0097 (9)	0.0100 (10)	0.0072 (9)	0.000	0.0047 (8)	0.000

### Geometric parameters (Å, º)

**Table:**

Pb1—O4	2.380 (3)	P1—O1	1.531 (2)
Pb1—O6^i^	2.5429 (18)	P1—O2	1.595 (3)
Pb1—O6^ii^	2.5429 (18)	P2—O4	1.535 (3)
Pb1—O3	2.7284 (17)	P2—O6	1.5432 (18)
Pb1—O3^iii^	2.7284 (17)	P2—O6^iii^	1.5432 (18)
Pb1—O5	2.846 (2)	P2—O5	1.554 (2)
Pb1—O1^iv^	2.857 (2)	O1—Co2^xi^	2.2302 (16)
Pb1—O2^iv^	2.952 (3)	O1—Co2^xii^	2.2302 (16)
Co1—O8	2.0544 (14)	O1—Pb1^iv^	2.857 (2)
Co1—O8^v^	2.0544 (14)	O2—Pb1^iv^	2.952 (3)
Co1—O3^v^	2.0624 (16)	O2—H2	0.8600
Co1—O3	2.0624 (16)	O5—Co1^xiii^	2.1766 (15)
Co1—O5	2.1766 (15)	O6—Co2^xiv^	2.1426 (17)
Co1—O5^v^	2.1766 (15)	O6—Pb1^ii^	2.5429 (18)
Co2—O7^vi^	1.9978 (14)	O7—Co2^xv^	1.9978 (14)
Co2—O7^vii^	1.9978 (14)	O7—Co2^xii^	1.9978 (14)
Co2—O6^i^	2.1426 (17)	O7—H7	0.8600
Co2—O6^viii^	2.1426 (17)	O7—H8B	1.6474
Co2—O1^ix^	2.2302 (16)	O8—Co1^xiii^	2.0544 (14)
Co2—O1^iv^	2.2302 (16)	O8—H8A	0.8600
P1—O3^x^	1.5274 (18)	O8—H8B	0.8600
P1—O3	1.5274 (18)		
			
O4—Pb1—O6^i^	82.12 (5)	O3^v^—Co1—O5	88.92 (8)
O4—Pb1—O6^ii^	82.12 (5)	O3—Co1—O5	91.08 (8)
O6^i^—Pb1—O6^ii^	97.00 (9)	O8—Co1—O5^v^	96.95 (6)
O4—Pb1—O3	105.34 (5)	O8^v^—Co1—O5^v^	83.05 (6)
O6^i^—Pb1—O3	171.63 (5)	O3^v^—Co1—O5^v^	91.08 (8)
O6^ii^—Pb1—O3	87.90 (7)	O3—Co1—O5^v^	88.92 (8)
O4—Pb1—O3^iii^	105.34 (5)	O5—Co1—O5^v^	180.00 (12)
O6^i^—Pb1—O3^iii^	87.90 (7)	O7^vi^—Co2—O7^vii^	180.0
O6^ii^—Pb1—O3^iii^	171.63 (5)	O7^vi^—Co2—O6^i^	87.93 (9)
O3—Pb1—O3^iii^	86.46 (8)	O7^vii^—Co2—O6^i^	92.07 (9)
O4—Pb1—O5	55.64 (7)	O7^vi^—Co2—O6^viii^	92.07 (9)
O6^i^—Pb1—O5	117.25 (4)	O7^vii^—Co2—O6^viii^	87.93 (9)
O6^ii^—Pb1—O5	117.25 (4)	O6^i^—Co2—O6^viii^	180.0
O3—Pb1—O5	65.72 (4)	O7^vi^—Co2—O1^ix^	100.06 (7)
O3^iii^—Pb1—O5	65.72 (4)	O7^vii^—Co2—O1^ix^	79.94 (7)
O4—Pb1—O1^iv^	132.09 (7)	O6^i^—Co2—O1^ix^	93.55 (8)
O6^i^—Pb1—O1^iv^	67.09 (5)	O6^viii^—Co2—O1^ix^	86.45 (8)
O6^ii^—Pb1—O1^iv^	67.09 (5)	O7^vi^—Co2—O1^iv^	79.94 (7)
O3—Pb1—O1^iv^	109.06 (5)	O7^vii^—Co2—O1^iv^	100.06 (7)
O3^iii^—Pb1—O1^iv^	109.06 (5)	O6^i^—Co2—O1^iv^	86.45 (8)
O5—Pb1—O1^iv^	172.27 (6)	O6^viii^—Co2—O1^iv^	93.55 (8)
O4—Pb1—O2^iv^	178.00 (7)	O1^ix^—Co2—O1^iv^	180.0
O6^i^—Pb1—O2^iv^	99.18 (5)	O3^x^—P1—O3	113.68 (14)
O6^ii^—Pb1—O2^iv^	99.18 (5)	O3^x^—P1—O1	112.68 (8)
O3—Pb1—O2^iv^	73.26 (5)	O3—P1—O1	112.68 (8)
O3^iii^—Pb1—O2^iv^	73.26 (5)	O3^x^—P1—O2	106.78 (8)
O5—Pb1—O2^iv^	122.36 (7)	O3—P1—O2	106.78 (8)
O1^iv^—Pb1—O2^iv^	49.91 (6)	O1—P1—O2	103.32 (13)
O8—Co1—O8^v^	180.00 (11)	O4—P2—O6	109.94 (9)
O8—Co1—O3^v^	87.37 (8)	O4—P2—O6^iii^	109.94 (9)
O8^v^—Co1—O3^v^	92.63 (8)	O6—P2—O6^iii^	107.30 (15)
O8—Co1—O3	92.63 (8)	O4—P2—O5	106.40 (14)
O8^v^—Co1—O3	87.37 (8)	O6—P2—O5	111.63 (9)
O3^v^—Co1—O3	180.00 (13)	O6^iii^—P2—O5	111.63 (9)
O8—Co1—O5	83.05 (6)	H8A—O8—H8B	104.5
O8^v^—Co1—O5	96.95 (6)		

Symmetry codes: (i) −*x*, *y*+1/2, −*z*; (ii) −*x*, −*y*, −*z*; (iii) *x*, −*y*+1/2, *z*; (iv) −*x*, −*y*, −*z*+1; (v) −*x*−1, −*y*, −*z*; (vi) *x*+1, *y*, *z*; (vii) −*x*, −*y*+1, −*z*+1; (viii) *x*+1, −*y*+1/2, *z*+1; (ix) *x*+1, *y*+1, *z*; (x) *x*, −*y*−1/2, *z*; (xi) *x*−1, *y*−1, *z*; (xii) −*x*, *y*−1/2, −*z*+1; (xiii) −*x*−1, *y*+1/2, −*z*; (xiv) −*x*, *y*−1/2, −*z*; (xv) *x*−1, *y*, *z*.

### Hydrogen-bond geometry (Å, º)

**Table:**

*D*—H···*A*	*D*—H	H···*A*	*D*···*A*	*D*—H···*A*
O2—H2···O6^ii^	0.86	2.28	3.027 (3)	145
O2—H2···O6^xiv^	0.86	2.28	3.027 (3)	145
O7—H7···O2^iv^	0.86	2.20	2.915 (3)	140
O7—H7···O3	0.86	2.53	2.986 (2)	114
O7—H7···O3^iii^	0.86	2.53	2.986 (2)	114
O8—H8*A*···O4^xv^	0.86	1.79	2.649 (3)	177
O8—H8*B*···O7	0.86	1.65	2.489 (3)	165

Symmetry codes: (ii) −*x*, −*y*, −*z*; (iii) *x*, −*y*+1/2, *z*; (iv) −*x*, −*y*, −*z*+1; (xiv) −*x*, *y*−1/2, −*z*; (xv) *x*−1, *y*, *z*.
